# Modern post-mortem imaging: an update on recent developments

**DOI:** 10.1080/20961790.2017.1330738

**Published:** 2017-06-07

**Authors:** Silke Grabherr, Coraline Egger, Raquel Vilarino, Lorenzo Campana, Melissa Jotterand, Fabrice Dedouit

**Affiliations:** a University Centre of Legal Medicine Lausanne-Geneva, Lausanne, Switzerland; b Service of Legal Medicine, Central Institute of Hospitals, Sion, Switzerland

**Keywords:** Forensic science, forensic imaging, post-mortem radiology, post-mortem computed tomography, post-mortem magnetic resonance imaging, 3D scanning, post-mortem angiography, photogrammetry

## Abstract

Modern post-mortem investigations use an increasing number of digital imaging methods, which can be collected under the term “post-mortem imaging”. Most methods of forensic imaging are from the radiology field and are therefore techniques that show the interior of the body with technologies such as X-ray or magnetic resonance imaging. To digitally image the surface of the body, other techniques are regularly applied, e.g. three-dimensional (3D) surface scanning (3DSS) or photogrammetry. Today's most frequently used techniques include post-mortem computed tomography (PMCT), post-mortem magnetic resonance imaging (PMMR), post-mortem computed tomographic angiography (PMCTA) and 3DSS or photogrammetry. Each of these methods has specific advantages and limitations. Therefore, the indications for using each method are different. While PMCT gives a rapid overview of the interior of the body and depicts the skeletal system and radiopaque foreign bodies, PMMR allows investigation of soft tissues and parenchymal organs. PMCTA is the method of choice for viewing the vascular system and detecting sources of bleeding. However, none of those radiological methods allow a detailed digital view of the body's surface, which makes 3DSS the best choice for such a purpose. If 3D surface scanners are not available, photogrammetry is an alternative. This review article gives an overview of different imaging techniques and explains their applications, advantages and limitations. We hope it will improve understanding of the methods.

## Introduction

Modern imaging techniques are of increasing importance in post-mortem investigations, especially in forensic and legal medicine, where such imaging techniques are most often used [1]. Their advantages are multiple: data can be stored digitally and accessed at any time allowing multiple image reviews; three-dimensional (3D) images can be reconstructed in an easily understandable format and used to explain complex cases to non-medical persons. However, besides those assets, the advantages and limitations of using digital imaging methods depend on the technique that is used [2], as the information that can be gained by an imaging exam is different for each method. For this reason, it is important to clearly define the techniques.

The use of radiological imaging methods for post-mortem purposes is nearly as old as radiology itself [3]. The first post-mortem images were developed shortly after the discovery of X-rays by Wilhelm Conrad Röntgen in 1895. To begin with this new method, which allowed assessment of the interior of objects and bodies, was mainly used in anthropology, and one year after Röntgen's discovery, in 1896, the mummified hand of an Egyptian princess was X-rayed [4]. Conventional X-ray imaging is still used regularly in legal medicine. Many institutes of legal medicine have a mobile X-ray unit that can be used to search for radiopaque foreign bodies or bone lesions inside the body. Classic indications for such X-ray exams are the examination of new-borns or babies to detect suspicious bone lesions, the imaging of putrefied or otherwise strongly altered bodies such as charred bodies or those hit by a train, as well as the imaging of completely unknown bodies with the aim of detecting dental or orthopaedic prosthetic material for possible identification [5]. Today, in forensic imaging, the most frequently used techniques are post-mortem computed tomography (PMCT), post-mortem computed tomographic angiography (PMCTA) and post-mortem magnetic resonance imaging (PMMR). In additional to these techniques coming from clinical radiology, 3D surface scanning (3DSS) is applied for forensic purposes and also for anthropological investigations. This paper shall give an overview of these four techniques and explain their applications, advantages and limitations (Table 1).

**Table 1.** Overview of advantages, limitations and typical indications of the most used methods in modern forensic radiology and 3D imaging.

Method	Advantages	Limitations	Typical indications
PMCT	– Short acquisition time – Relatively easy handling – Ideal for 3D reconstructions – Excellent visualization of skeletal system, gas, foreign bodies, fluids – Relatively low architectural equipment necessary – High spatial resolution	– X-rays – Low contrast in soft tissue – Formation and training necessary for interpretation of images – Relatively high maintenance costs – High volume of data needs special equipment for storage	– Trauma, especially skeletal system trauma (accidents, fall from height, traffic accidents, blunt force trauma) – Ballistic trauma – Child abuse – Detection of foreign bodies – Identification – Skeletal age estimation – Detection of air/gas (e.g. air embolism) – Changes of skeletal system
PMCTA	– Good soft tissue contrast – High spatial resolution – Ideal for 3D reconstructions of the vascular system – Method of choice for detecting bleeding sources – Minimally invasive	– Time consuming – Needs preparation of the body – High volume of data needs special equipment for storage – Formation and training necessary for interpretation of images – Costs for injection material/contrast agent	– Trauma, especially vascular system trauma (accidents, blunt force trauma, sharp trauma, ballistic trauma) – Vascular pathologies in natural death (pathological changes in coronary arteries, systemic vascular diseases) and vascular anomalies – Death after surgical/medical intervention
PMMR	– No X-rays – Excellent soft tissue contrast – High spatial resolution	– Long acquisition time – Complex handling – Very high maintenance costs – Special architectural conditions needed – 3D reconstructions need special sequences – Training/formation for interpretation of images needed – High volume of data needs special equipment for storage – Danger with ferromagnetic foreign bodies	– Traumatic organ lesions (blunt trauma, sharp trauma) – Organ lesions due to systemic of local illnesses (natural death) – Strangulation – Child abuse – Skeletal age estimation
3D Surface documentation	– Excellent visualization of surface – High spatial resolution (3DSS) – Ideal for “3D modelling” and reconstruction – Low maintenance costs – Mobile	– Time consuming (3DSS) – Complex handling (3DSS) – No information about inner findings – Data treatment needs specialist	– Trauma (traffic accidents, blunt force trauma) – Reconstructions of traffic accidents, crime scenes – Comparison between injuries and objects – Comparison between bite marks and dentition – Digitalization of objects (e.g. bones, murder weapon, etc.)

Abbreviations: PMCT: post-mortem computed tomography; PMCTA: PMCT-angiography; PMMR: post-mortem magnetic resonance imaging; 3DSS: 3D surface scan.

## PMCT

Since the first report of an unenhanced PMCT scan in 1983 [6], the method has become an important additional tool for the conventional autopsy [7–13]. This technique is based on the different degrees of tissue attenuation of X-rays from a rotating source coupled to detectors that transmit the data to a computer that uses various algorithms to generate tomographic or 3D images. The brightness of a given tissue depends on its attenuation of X-rays. For forensic purposes, the body is partially (only head or thorax) or entirely (from head to toe) scanned. The obtained images may be viewed in any plane or as 3D reconstructions. These are widely used to show findings to non-medical experts, particularly in cases of skeletal trauma [14]. Although such reconstructed 3D images are highly explanatory and easily understandable, particular attention must be paid to the fact that they are not ideal models for detailed analysis, as they provide only reconstructed images and their precision depends on many different factors such as the scan parameters, the presence of metallic particles and other sources of artefacts.

The performance of routine whole-body PMCT is a current procedure in many forensic institutes with their own multi-detector computed tomography (MDCT) units. Many other centres have access to clinical MDCT units located within the hospital's radiology department. The data are recorded and available indefinitely, long after the body has been interred or cremated. So far, most published studies have shown the importance of PMCT as a tool for qualitative improvement in forensic pathologic investigation, without banishing the conventional autopsy that provides information about organ morphology [7–13].

**Figure 1.**
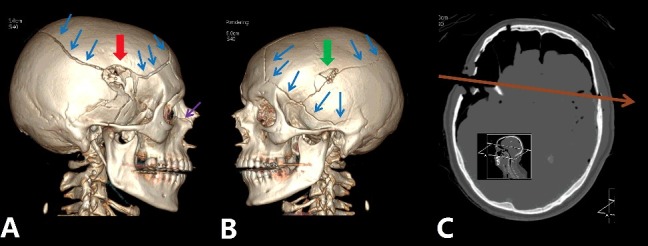
Demonstration of ballistic trauma by PMCT using 3D-volume rendering reconstructions (A and B) and a paraxial axial image. (C) Visualization of multiple fractures of the skull (blue arrows in A and B) with an entrance wound (red arrow in A) in the right temporal region and an exit wound (green arrow in B) on the left side. The trajectory of the bullet is given in the axial oblique reconstruction (brown arrow in C).

PMCT is useful in trauma cases, which are the cases most often examined using this method [15,16]. PMCT rapidly depicts the presence of bone fractures, which are important information not only for defining the cause of death, but also for medico-legal case reconstructions. It is very useful in cases of blunt force trauma [17] (traffic accidents, air plane crashes or falls from heights) and ballistic trauma [14,18]. 3D reconstructions of the fractures help to understand the fragment relationships and can be used, for example, in court, as they are less unpleasant than conventional autopsy images. They are also useful in indicating gunshot trajectories in ballistics cases (Figure 1).

Similarly to conventional X-ray imaging, PMCT allows the localization of radiopaque foreign material. In medico-legal cases, it is used to detect ballistic foreign bodies, mostly projectiles [14,18]. It is used in the investigation of body packers (living or deceased) [19,20], searching for medical devices for identification purposes (prosthetics or dental implants) and especially in cases of decomposed bodies. Such images can be used to simply show the radiopaque foreign material and depict its position in the body, or to guide the forensic pathologist in extraction during the conventional autopsy.

In cases of infant death, if magnetic resonance imaging (MRI) is not possible for technical, or practical reasons (no MR unit accessible, personnel for MRI not available), PMCT is mandatory if available, as skeletal injuries or malformations are often difficult to see in a conventional autopsy [21,22]. Also, it must be noted that radiological examination of infant bodies can be used in non-forensic cases as the parents frequently will not agree to an autopsy.

For the detection of blood that may indicate haemorrhage (e.g. haemopericardium, haemothorax, haemoperitoneum, cerebral bleeding, etc.), performing a PMCT is enough, but if the exact location of the bleeding source is questioned, a PMCTA may also be performed. In cases of medical malpractice or after a medical intervention, especially in cardiac cases [23], PMCT can detect the presence of haemorrhages and gas or air in the vessels, and should be followed by a PMCTA for a complete examination.

Because of its very low attenuation, gas or air is easily recognized on PMCT, allowing detection of small amounts in soft tissues or anatomic cavities. Correct interpretation of the origin of the gas may be more challenging. Several studies have shown that formation of post-mortem gas begins during the first hours after death [24], which makes interpretation of its origin difficult. To interpret accurately, it is important to know the usual distribution of post-mortem gas [25]. This is why the only way to be certain about its origin is the chromatographic analysis of its composition. Therefore, methods using CT-guided gas sampling have been established [26], which can be used for a medico-legal case if, for example, a diagnosis of vital air embolism is suspected [27].

In some centres, studies have investigated the ability to increase the sensitivity of PMCT for pulmonary findings by performing CT data acquisition during pulmonary ventilation [28–31]. Post-mortem ventilation is used to mimic clinical CT scanning performed during breath holding in living individuals. Different devices have been proposed for ventilation: endotracheal tubes, laryngeal masks and supraglottic airways. While the application in post-mortem cases differs according to the handling in rigid bodies, the results are promising.

PMCT has limitations in its visualization of the organ parenchyma and vascular system. This makes the technique especially limited in cases of natural death. For example, in cases of sudden cardiac death, the cause of death cannot be verified by PMCT alone. Although it can give information about ischemic heart disease (IHD) by detecting the presence of calcifications in the coronary arteries, it can only raise the suspicion of pathological changes in the heart. In these cases, PMCTA is more useful for the diagnosis of IHD [32,33].

A new development in MDCT is the dual-energy computed tomography (DECT) technique. While conventional or single-energy CT uses a single polychromatic X-ray beam, emitted from a single source, DECT uses two different energy X-ray beams. This additional energy level can generate an additional data-set during scanning. Such DECT units are potentially useful for forensic purposes, as they allow differentiation of tissue densities and reducing artefacts in the obtained images [34–36]. Few studies have been performed that investigated the application of this technique for forensic purposes [37], especially for differentiating drugs in cases of body packers [38].

## The method

As mentioned earlier, the method has some limitations restricting interpretation of images such as, for example, the absence of tissue contrast in soft tissues or parenchyma of internal organs. In a deceased, vessels are often flattened, obscuring visualization of their lumen. CT angiography is helpful in these cases.

Based on clinical radiology experience and development, several research groups have been created among the medico-legal communities with the aim of developing a method for post-mortem angiography using a contrast agent to better distinguish and clearly visualize the characteristics of soft tissues and inner organs, as well as the vascular system [30]. Planar post-mortem angiography techniques already exist in the literature but most have only been applied directly after death and in single organs [39–41]. Most of those methods were used at the end of the nineteenth and the beginning of the twentieth century. The types of injected substances can be divided into six groups: vascular casts, corpuscular preparations in gelatine or agar, corpuscular preparations in aqueous solution, soluble contrast material, oily contrast material and miscellaneous formulations [40]. However, to apply PMCTA in the context of modern forensic and legal medicine, none of the classic techniques seem to be adequate, as they are only applicable shortly after death, require the removal of the surrounding soft tissue or can only be performed on isolated organs [40]. For this reason, new investigations have been opened to develop methods that could be applicable in the context of modern post-mortem imaging.

Several post-mortem angiography techniques have been tested [42]. In Switzerland, the Virtopsy® group in Bern investigated opacification of the vessels of the whole body with a clinical contrast agent mixed with polyethylene glycol (PEG) [43]. In England, where the investigation of cardiovascular death is a main indication for autopsy, localized injection of contrast agent, the so-called targeted coronary angiography, was developed as a technique, which focuses on pathology of the myocardium and coronary arteries [44,45]. The coronary arteries are opacified with contrast agent injected directly into the ascending aorta through a cannula in the subclavian or carotid arteries. In France, CT angiography has been described in case reports [46]. Ultrasound imaging was employed to guide the insertion of catheters into the vessels [46]. In Japan, the technique of PMCTA using manual chest compression was developed [47,48]. This method is especially adapted to local conditions where the body can be directly investigated after death in the clinical environment. It consists of the injection of a contrast agent into peripheral veins such as the cubital vein. The contrast agent is then transported through the body with active chest compression of the deceased on the CT table. After about 2 minutes of chest compression, a CT scan is performed.

In Lausanne, Grabherr et al. developed multiphase post-mortem CT angiography (MPMCTA) [49] with the goal of establishing PMCTA as a routine technique for forensic medicine with standardized procedures and equipment. MPMCTA is the most widespread and studied technique of post-mortem angiography. It is a minimally invasive technique which allows complete filling of the vessels of the head, thorax and abdomen. The first step is an unenhanced PMCT, which is then followed by the cannulation (Virtangio® Tubing Set, Fumedica AG, Muri, Switzerland) of the femoral artery and vein on one side of the body. These vessels are accessed using a short incision in the inguinal region. The two cannulas are connected to a perfusion device, especially conceived for post-mortem angiography (Virtangio® Machine, Fumedica AG).

Different injection materials were tested and the one selected was an oily liquid based on its ability to remain intravascular, which enables the investigation of even putrefied bodies if a post-mortem delay of the investigated body occurs [50,51]. A specific oily contrast agent has been developed (Angiofil® Macro, Fumedica AG) and is used as a mixture with paraffin oil [52]. Because of its viscosity, the capillary system, which is the most vulnerable to post-mortem changes, is not perfused and the contrast medium enters the venous system via arterio-venous shunts, without inducing oedema in soft tissue or extravasating into body cavities. It does not mix with any remaining blood, so that haemorrhages can still be quantified by measuring blood volume during autopsy.

The MPMCTA method consists of four phases: First, an unenhanced PMCT is done, then, during and after the contrast agent injection, three angiographic phases are performed: an arterial, a venous and a dynamic phase. A study based on 45 human bodies enabled the establishment of a standardized perfusion protocol [49]. Grabherr et al. concluded that to be significant, a finding must be visible in at least two phases, otherwise it is considered an artefact [49]. The advantages and limits of MPMCTA were investigated with a study based on 50 autopsy cases including conventional medico-legal autopsy, PMCT and MPMCTA [7]. The authors concluded that the combination of MPMCTA and autopsy provided the best results, and helped to increase the quality of post-mortem investigations.

The aim of all PMCTA techniques is to perform vascular diagnosis similar to clinical investigations, meaning to identify the source of haemorrhage [53], the quantification of blood loss and the detection of anatomical variations and pathological abnormalities of the vascular system. PMCTA enables the same handling of acquired image data as in clinical radiology. This includes, for example, the 3D reconstruction of vessels, such as the coronary arteries. This is especially important considering the high number of cases of sudden cardiac death in the daily routine of the forensic pathologist [54,55]. Indeed, with conventional autopsy, visualization of vessels is extremely difficult and time consuming, sometimes even impossible, especially where small vessels are concerned. MPMCTA is suitable to estimate vascular stenosis and/or occlusion [32], as well as to determine the patency of a graft. It allows evaluation of the entry and extension of a vascular dissection into other vessels and easily visualizes vascular anomalies, such as anatomical variations.

**Figure 2.**
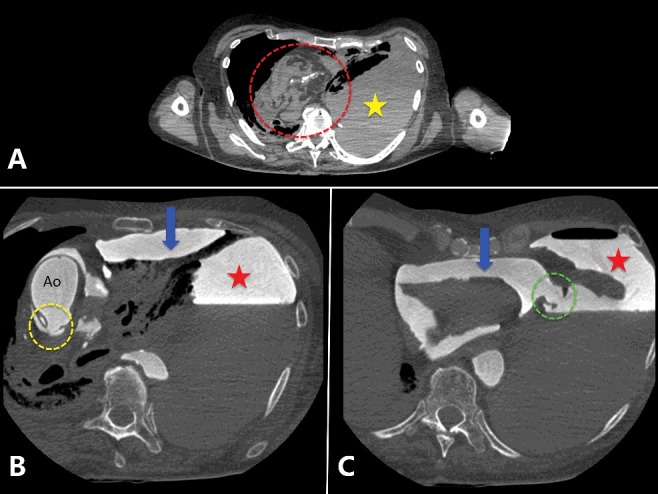
2D axial cross-sectional images obtained in a case of sudden death of an 87-year-old man, who suddenly fainted in presence of witnesses, with unsuccessful cardiopulmonary resuscitation.

Post-mortem angiography is also recommended in cases of traffic accidents, falls from heights, suspected cardiovascular deaths (Figure 2), fatal outcomes of medical or surgical intervention [56], sharp force trauma and ballistic trauma [57,58]. In cases of gunshots, the path of the bullet through soft tissues and organs is highlighted by the extravasating contrast agent and can be easily shown to the court and understood by non-medical workers, while the exact location of projectiles is already documented by non-contrast PMCT. In cases of cardiovascular death, MPMCTA not only allows analysis of the coronary arteries but also provides more detailed information about the myocardium, accumulating in infarcted myocardium [59], guiding the autopsy and histological sampling [54].

MPMCTA-induced artefacts are well known, such as the artefactual leakage of contrast agent around the head of the pancreas and throughout the gastric mucosa into the stomach in cases where autolysis has begun [60].

MPMCTA has known limitations, such as in the diagnosis of pulmonary thromboembolism, which can be ruled out when a pulmonary artery shows no perfusion defect, but can only be suspected when a filling defect is noted in the vessel. This defect can be due to a pulmonary embolism but also due to large post-mortem clots, which tend to be present in these vessels [60].

The standardized protocol of MPMCTA, with cannulation of femoral vessels on one side of the body, hinders visualization of the vessels of lower limb beyond the cannulation site. Therefore, if the question of investigating the vessels of the leg arises, an axillary approach is proposed to enable detection of thrombosis of superficial and deep venous systems in the lower extremities [61].

Another limitation of MPMCTA is that the oily contrast agent mixture mimics fatty pulmonary embolism. In a polytrauma situation, for example, when a forensic diagnosis of vitality of the trauma is required, CT-guided pulmonary tissue sampling using clinical biopsy needles can be performed before the injection of the oily contrast agent to allow reliable histological samples [49,62]. It is recommended to allow microbiological and toxicological analysis samples to be taken with or without CT-guidance prior to the injection of the contrast agent [63,64].

MPMCTA is standardized for the application in adult bodies. Paediatric protocols will be developed in the future [65,66].

## PMMR

In contrast to PMCT and PMCTA, MRI is a technique that does not use any radiation. Strong magnetic fields and radiofrequency waves are used to influence the spins in the tissue to be depicted. An “echo” of the spin excitation, depending on the time spins take to return to their original state after excitation, is recorded and used to calculate the images. The recorded signal intensity for each voxel is assigned a grayscale value to visualize intensity differences. Biochemical and physical properties of tissues strongly influence spin relaxation times and thus lead to excellent tissue contrast [5].

Although MRI is widely used in clinical medicine, the routine diffusion of this modality into forensic medicine has been limited [67]. PMMR is still underutilized in forensic pathology, although it is a powerful diagnostic tool for forensic radiology [68]. This can be explained by the more limited access to MR scanners due to time constraints in clinical radiology, and by the expense and the complexity of MR technology. In fact, to obtain satisfying results, acquisition protocols have to be adapted which have to balance between the field of view and the obtained resolution (the smaller the field of view, the better the resolution). Additionally, the user should consider an ideal slice orientation for the resulting images. Unlike CT, images are usually acquired in one plane at a time. The examination time that is needed to perform high-quality PMMR is an important limiting factor. For these reasons, PMMR is mostly performed in one anatomic region of specific interest and cannot be used as a screening method like PMCT. To perform a PMMR, information is also needed about the presence of indwelling ferromagnetic foreign bodies. As MRI employs high magnetic field strengths, such foreign bodies can be dislodged. Any metal on the body presents a risk of being a projectile, which is the reason why each body has to be inspected before it is placed in an MRI scanner.

PMMR is subject to artefacts, some specific to PMMR [59]. Vascular stasis and, in particular, venous stasis may mimic haemorrhage on T2-weighted sequences. The presence of gas may also modify the quality of the images, as it produces a signal void. The influence of temperature on MR image contrast occurs in T1- and T2-weighted images, with changes natural tissue contrast [69]. The absence of circulation renders impossible the use of dynamic clinical MR sequences such as (arterial or venous) Time of Flight imaging affords information concerning potential vascular occlusions, dilatations (aneurysms, ectasia) and stenoses without contrast medium injection. The ideal protocol consists of T1- and T2-weighted sequences, acquired in three different axes or with special 3D acquisition and multiplanar reconstruction or maximum intensity projections.

The ability of MR images to provide anatomical information, and to highlight fluid accumulations, makes it an ideal diagnostic tool for a wide range of pathologies, including subcutaneous haematoma, bone contusion, organ laceration, internal haemorrhage and fluid collections, ischemic injury of the heart, brain oedema, pericardial or pleural effusion and pulmonary oedema [68].

An important interest in PMMR research is cardiovascular imaging. Acute, chronic and even subacute infarction are visible on PMMR [70]. The post-mortem imaging findings of acute myocardial infarction are comparable to those found in clinical cardiac MR and consist of focal necrosis surrounded by perifocal myocardial oedema with increased signal intensity on T2-weighted images. Focally decreased signal intensity within the myocardium on T2-weighted images without perifocal oedema was interpreted as a sign of early acute myocardial infarction (with a survival time ranging from minutes to hours) [70]. Due to the excellent tissue contrast, some measurements of thickness of the myocardium can be made, which allows identification of left ventricular hypertrophy.

**Figure 3.**
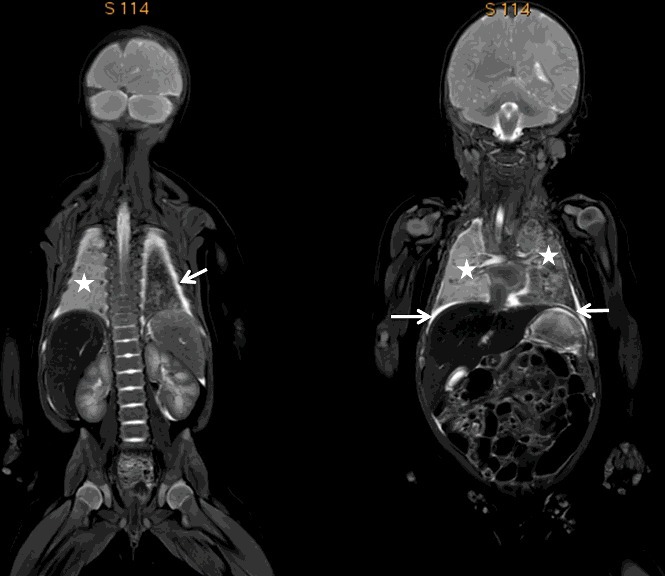
Coronal total body T2-weighted images of a 3-month-old female in the context of SIDS (sudden infant death syndrome) showing slight bilateral pleural effusions (white arrows) and bilateral lung parenchyma consolidations (white stars). The morphology of the abdominal organs is easily identified.

High tissue contrast and the ability of MRI to visualize soft-tissue pathology are also the principal reasons why PMMR is the modality of choice in post-mortem neonatal and paediatric imaging (Figure 3) [71]. Concerning foetal PMMR, the literature is mainly non-forensic. MRI is an alternative to determine potential causes of death when handling non-legal cases with parental objection to autopsy. The MRI Autopsy Study (MaRIAS) was the first large prospective study to evaluate the clinical usefulness of PMMR as an alternative to full conventional autopsy in foetuses and children [72]. A total of 400 unselected cases were analysed, which included 277 (69%) foetuses and 123 (31%) children younger than 16 years. The MaRIAS clearly showed the advantages of PMMR for paediatric post-mortem imaging [73].

The use of post-mortem contrast in MRI, similarly to PMCTA, is called PMMR angiography (PMMRA). Ruder et al. reported whole-body PMMRA using clinical aqueous iodinated contrast medium diluted in a PEG solution [74]. Good image contrast was obtained using fat-saturated T1-weighted images. As suggested by Ruder et al. and based on our personal experience, we suggest that the time between contrast injection and imaging should be minimized [50]. Recently Bruguier et al. developed a protocol using an oily contrast agent for PMMRA on *ex situ* hearts permitting high-quality images and visualization of the coronary arteries [75].

Another MRI technique, diffusion-weighted imaging (DWI), evaluates the diffusion of water in tissues [76]. Changes in water diffusion can be quantified by DWI and are routinely used in clinical medicine for the early diagnosis of stroke.

Some authors have assessed the utility of an MRI technique called proton magnetic resonance spectroscopy to characterize time-dependent cerebral metabolic changes in sheep [77]. This chemical study of the post-mortem decomposition of brain tissue was thought to potentially estimate the post-mortem interval.

As there is no radiation in MRI, it can also be used for forensic purposes on living persons. In clinical forensic medicine, some authors have suggested its use in non-lethal strangulation cases to document life-threatening strangulation. This technique can reveal intramuscular haemorrhage and oedema, swelling of the platysma, intracutaneous bleeding, subcutaneous bleeding and haemorrhagic lymph nodes [78–80]. It is also useful in child abuse cases, in particular to diagnose shaken baby syndrome, where it can document peri-cerebral lesions [81]. A precise datation of those lesions is a myth and dating the incident, however, remains controversial [82]. Recent publications have emphasized that MRI of the spinal column should be considered in any child with suspected abuse and head trauma [83,84] as there is clear evidence of spinal involvement in a proportion of cases of abusive head trauma.

Many publications have discussed the use of MRI for bone age assessment [85]. Due to its optimal tissue contrast, MRI allows examination of the cartilage, which cannot be done with MDCT. The progressive maturation of the epiphyses and the changes of the growth plate cartilages with age are well known [86]. The use of MRI to document this phenomenon has led to multiple studies investigating regions including the teeth, clavicle, wrist, iliac crests, knee and ankle [87–98].

Compared with PMCT, PMMR seems to be in its early stages of introduction into post-mortem imaging. Special protocols may be extremely useful for forensic purposes and many new developments can be awaited in this field during the coming years.

## 3D surface documentation

Data obtained by cross-sectional imaging techniques can be used to reconstruct 3D models of the body's surface. However, although the spatial resolution in post-mortem imaging is relatively high compared with clinical imaging, the resolution of such 3D models is mostly limited. Therefore, lesions like small abrasions of the skin cannot be documented by using these radiological techniques. Also, information about the colour of the lesion is missing. To add this information about the body's surface to the digital data in a manner that allows forensic reconstructions, other imaging techniques which are better adapted to document the surface of objects or the skin are therefore necessary.

### Photogrammetry

During the last decade, 3DSS has begun to play an important role in forensic medicine. Prior to the advent of 3DSS, 3D photogrammetry techniques were applied to supply 3D models of, for example, an injury [99,100]. These techniques allowed generation of a 3D model from an object after having merged different photographs taken from different sides and angles. They were, however, complicated and time consuming, as for every 3D point obtained from the model, identical points had to be found manually on every image to be inserted. As an aid, a grid projection was projected upon the object during the photogrammetric capturing to facilitate the confirmation of more identical points in every image [99,100].

Recent new software technologies have made it possible to construct 3D models based on photogrammetry much more easily and almost fully automatically. Instead of using specific 3D surface scanners [101], it is possible to use any digital camera to capture the object to be represented in 3D. To obtain better results, a high resolution SLR camera is recommended. Respecting some camera parameters to obtain sharp images of the object, the only thing needed to create a 3D model is pictures taken from different angles by maintaining more or less the same distance to the object. This distance is chosen according to the size of the object, as each image should be completely represented, and so as to obtain better results for the 3D model, images should also be overlapping (ideally 80%). The object should fill the entire frame in order to avoid any background. It is necessary to put at least one scale bar or a ruler on or nearby the object, which is then visible in several images. This allows the object data to be stored correctly.

After the capture process, all image data are imported into 3D software that will generate the 3D model step by step [102]. The first step is a fully automatic alignment of the images. As the software recognizes several identical natural contrast variations in the pixels of different images, it is able to link them and to calculate a bundle alignment. Once the images are oriented in this way, a dense point cloud can be calculated, which is used as a next step to build a mesh. Further, a texture for the 3D model can be designed by using all the captured images. This step allows the 3D model to get its realistic colours. Finally, the dimensions of the object should be defined by marking the ends of the scale bar or ruler in different images.

The 3D resolution of the models related to this method depends on different parameters [103]. However, the method is inferior to 3DSS, especially the high resolution structure light scanners. Still, for some forensic cases, this resolution may be sufficient, as in many cases the true colour of an injury is more important than the resolution in 3D geometry. This true colour is what the new photogrammetry technology offers. However, a disadvantage of this technology is that it does not allow checking if the acquired images are employable to the 3D software or not until the whole data-set has been transferred into a 3D software program. This is different to 3DSS where the resulting 3D model can be viewed in real time while scanning. Nevertheless, this low cost method of 3D documentation offers a good alternative, if 3DSS scanners are not available.

### 3DSS

**Figure 4.**
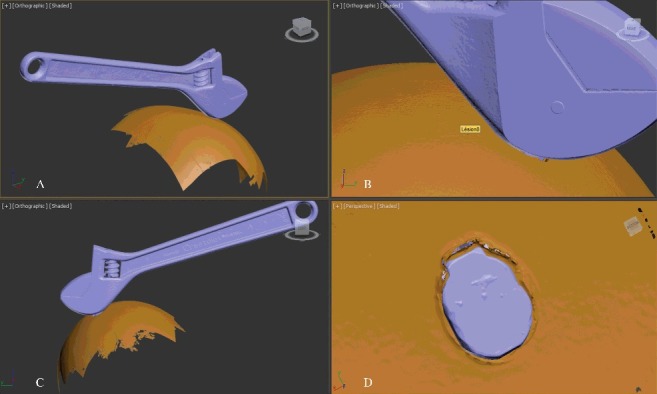
Comparison of 3D models of the surface of an object to a “lesion” obtained by a GOM-Atos fringe light surface scanner. A mark was created on the surface of a watermelon in the context of a research project (A--C). The mark or “lesion” (D) is compared to the surface of the suspected injury-causing object (here a wrench).

3DSS is a technique that is regularly used for forensic purposes in several institutes of legal medicine and in police services, especially in Switzerland. It is employed for reconstructions of traffic accidents [104] or to correlate injuries with the suspected injury-causing instrument [105]. Typically, it is used to compare a bite mark on a victim to the dentition of the suspected perpetrator [106]. The scanners used for forensic purposes are so-called “fringe light scanners”. They consist of one projector and two cameras. During a scan, a fringe light is projected onto an object and moved over its surface. This movement leads to the deformation of the fringes that is registered by the cameras. The computer that is linked to the scanner is capable of calculating 3D coordinates on the surface of the object. The calculation of this point cloud is based on the principle of triangulation. High performance scanners can calculate up to 16 million points per scan on the surface of an object. Complex objects have to be scanned from different angles and different distances. Similarly to photogrammetry, the computer merges together the different scans for rendering the 3D model more and more complete. At the end of the digitization process, a 3D model of high resolution can be obtained. The exact resolution depends on the type of scanner and can vary significantly. In general, mobile hand scanners are practical to be moved, for example, to a crime scene, but the best resolution is obtained using static scanners. Most performing scanners reach a spatial resolution of 0.017 mm [5]. To compare a lesion to the object that is suspected of having caused the damage, the 3D models can be superimposed (Figure 4). Different software systems are available for this purpose.

All data from 3D surface documentation can be merged with other imaging data such as CT or MRI. This allows complex medico-legal and forensic reconstructions such as the reconstruction of a crime scene or a traffic accident [104]. According to the users of the technique, multiple advantages can be observed. In fact, the technique is non-invasive, permits a more or less simple digitization (depending on the scanner that is used) and allows the creation of high-resolution models. Although 3DSS is usually more time consuming that photogrammetry, it is still a relatively rapid technique, as most modern scanners can digitize an object immediately, allowing a scan of a whole body in about 15 minutes. Despite these advantages, it is important to emphasize that the technique was not originally developed for forensic purposes, and therefore, the available software may be inadequate in some situations. For example, the scanners were developed for the production of optimal images of smooth surfaces. They were not constructed for scanning a surface such as human skin [107]. Also, scanning a dark or reflecting surface can be difficult. Although it is stated in many papers that the technique is “objective and easy”, the result strongly depends on the experience of the user. Further, most scanners are extremely sensitive to movements or changes in light, rendering outdoor use nearly impossible.

Despite the abovementioned limitations, the use of 3D documentation techniques in forensic medicine is important and 3D imaging is a powerful tool. It has proved itself in many cases of legal medicine and has determined medico-legal investigations in court. If correctly used, this technology can be the key for solving questions of complex medico-legal reconstructions. Additionally, the market for 3D imaging tools is developing rapidly, leading to the hypothesis that these methods will gain even more importance in the near future.

## Conclusion

Today, many different techniques exist to obtain digital images of the body and its surface. These techniques, which can be collected under the heading “post-mortem imaging”, have different advantages and drawbacks. They all allow digitization of data; each modality visualizes different parts of the body optimally, including the skeletal system (PMCT) the vascular system (PMCTA), soft tissues (PMMR) or the body's surface (3DSS and photogrammetry). By using them together on one case, most parts of the body can be investigated. By applying only one of the techniques, the images obtained are subject to the limitations of that modality.

Today, imaging techniques are mostly used independent of one another in combination with conventional autopsy. They are chosen according to the particulars of a case in order to increase the sensitivity of the post-mortem exam. While the existing technology is impressive concerning resolution and rapidity of most exams, the most important challenge today remains the correct interpretation of the images. Therefore, efforts have to be made concerning teaching and training specialists in the field.
